# Editorial: Single cell intelligence and tissue engineering

**DOI:** 10.3389/fbioe.2022.1019929

**Published:** 2022-09-06

**Authors:** Jiaofang Shao, Yangzi Jiang, Zhaoyuan Fang

**Affiliations:** ^1^ Department of Bioinformatics, School of Biomedical Engineering and Informatics, Nanjing Medical University, Nanjing, China; ^2^ Institute for Tissue Engineering and Regenerative Medicine, School of Biomedical Sciences, Faculty of Medicine, The Chinese University of Hong Kong, Shatin, Hong Kong SAR, China; ^3^ School of Biomedical Sciences, Faculty of Medicine, The Chinese University of Hong Kong, Shatin, Hong Kong SAR, China; ^4^ Department of Orthopaedics and Traumatology, Faculty of Medicine, The Chinese University of Hong Kong, and Prince of Wales Hospital, Shatin, Hong Kong SAR, China; ^5^ Center for Neuromusculoskeletal Restorative Medicine, Shatin, Hong Kong SAR, China; ^6^ Zhejiang University-University of Edinburgh Institute, Zhejiang University School of Medicine, Haining, China; ^7^ The Second Affiliated Hospital, Zhejiang University School of Medicine, Hangzhou, China

**Keywords:** single-cell, computational methods, biomarker identification, clinical application, profiling, machine learning

Single-cell sequencing has emerged as a powerful technology to dissect the heterogeneity of complex biological tissues at genomic, epigenomic, and transcriptomic levels, and has been extensively applied in various biological researches particularly in disease mechanisms and developmental biology (Paik et al., 2020; Gohil et al., 2021; Lei et al., 2021). Since the first single-cell RNA-sequencing (scRNA-seq) publication in 2009 (Tang et al., 2009), single-cell-based technologies have generated massive datasets, offering great opportunities to fully address biomedical problems as well as posing a challenge to computational analysis. At the same time, machine learning methods have been successfully used in processing many kinds of big data, including scRNA-seq data analysis (Petegrosso et al., 2020; Flores et al., 2022).

Nevertheless, more in-depth studies by using elegant methods and strategies to analyze the massive data obtained from the sequencing are still in need to improve our understanding of complex disorders. To make the best use of the single-cell-based data, researchers would first demand efficient and accurate computational pipelines to cluster, annotate cell types, uncover the marker genes and perform functional analysis. Besides, proper study design, including dataset selection and cross-validation, should be conducted to ensure that the evidence is convincing. In this context, this research topic included nine research articles focusing on methods development and clinical application of single-cell technology, giving more examples of data analysis and application in biomedical research.

One of the most common applications of single-cell approaches is to identify and distinguish cell types, and more related computational methods are demanded. Li et al. trained several classifiers and obtained optimal models from *in vitro* cultured human hepatocyte single-cell RNA data, and identified biomarkers for distinct differentiated hepatic cell types. By uncovering qualitative features for different stages of differentiation of liver cells, this study aimed to provide potential targets for cell transplantation to treat liver diseases. Similarly, Li et al. applied several different machine learning methods to expression profiling data of human pancreatic islet cells at single-cell resolution from both type 2 diabetes (T2D) patients and non-diabetic donors and discovered several T2D-associated genes. These two studies showed the promising applications of machine learning using single-cell expression profiling datasets to understand complex diseases.

Clustering is a critical step in single-cell data analysis to reveal heterogeneities and recognize cell types, requiring efficient and accurate computational algorithms. Tian et al. utilized an enhanced consensus-based clustering model and developed a novel computation method scMelody to cluster cells with single-cell DNA methylation data, such as scME-seq, scBS-seq, scWGBS, scTrio-seq, scNOMe-seq and snmC-seq. By using seven real single-cell methylation datasets and a variety of simulated datasets with different initial settings, scMelody showed better clustering performance and scalability when compared to other existing methods. In the two case studies, scMelody was able to uncover novel cell clusters from human hematopoietic cells and mouse neuron datasets.

Another two studies focused on developing prediction models for diagnosis using machine learning methods. Wang et al. explored mutation signatures with pan-cancer whole exome sequencing data, and constructed a logistic regression model to distinguish cancer types. The proposed model was able to trace the tumor origin for metastatic cancers, and predict cancer types using plasma ctDNA. Wu et al. investigated the microbiota in lung tissue and bronchoalveolar lavage fluid from lung ground-glass opacity (GGO) patients, and constructed a model using 10 genera-based biomarkers to predict GGO.

In addition to the biomarker identification for disease diagnosis, it has been widely recognized that the cellular heterogeneity is prevalent in either tissue or cultured cancer cells. Li et al. first identified a shared sub-cluster cancer stem cells (CSC) using 4 scRNA-seq datasets from upper gastrointestinal cancer (UGIC) patients including head and neck squamous cell carcinoma (HNSCC), esophageal cancer (EC), and gastric cancer (GC), and then compared the specific cells to scRNA-seq datasets from other 6 cancers including glioma, melanoma, osteosarcoma, breast cancer, ovarian cancer and stellate cell cancer to validate the specificity. The UGIC-specific CSC upregulated 33 genes while downregulated 141 genes compared to other tumors analyzed in this study, involving in inflammatory and Wnt pathways. Smit et al. applied FUNseq to spatially profile human breast epithelial cell line MCF10A which is a widely used *in vitro* model for breast cell transformation at single-cell resolution to decipher intratumor heterogeneity. By comparing the gene expression profiles and cell-cell communication among the cell populations at outer, middle and inner regions in 2D culture, the researchers found that cells at the outermost edge are most invasive, with epithelial-to-mesenchymal transition strongly activated.

While the advent of scRNA-seq technology has enabled profiling gene expression for each cell, analyses of large sample cohorts are still limited. The integration of existing bulk transcriptomic datasets is one of the issues that deserve attention and discussion in data mining. By taking advantage of the power of single-cell approaches and the sample size of bulk methods, two studies provided novel insights for diseases. Shi et al. collected a series of single-cell transcriptome datasets from non-failure hearts, dilated cardiomyopathy and ischemic cardiomyopathy hearts, followed by a comprehensive analysis to find out key genes involved in heart failure. Furthermore, the researchers obtained bulk gene expression datasets to validate the findings from single-cell datasets. On the other hand, Yao et al. developed a practical deconvolution pipeline by constructing a signature gene matrix which was then used to estimate cell proportion from bulk data with CIBERSORTx. Using preeclampsia microarray data, the researchers found that the proportion of trophoblast cells might contribute to the pathogenesis of the disorder.

As discussed above, articles in this special issue covered the fields of single-cell intelligence analysis methods development and computational model construction, providing more options for utilizing clinical datasets. By employing single-cell transcriptome data from patients, the researchers demonstrated the power of single-cell technology in important cell type recognition and key gene identification, broadening the clinical application of the technology. We envision that the use of advanced computational analysis approaches in single-cell datasets will reveal more useful and accurate biomarkers, and greatly benefit the diagnosis and treatments of complex diseases.
